# The Spectrum of Peripheral-Vestibular Deficits and Their Change Over Time in CANVAS/RFC1-Related Ataxia Systematic Review and Meta-Analysis of Quantitative Head-Impulse Testing

**DOI:** 10.1007/s12311-025-01825-y

**Published:** 2025-03-20

**Authors:** Maja Szymanska Heydel, Felix Heindl, Annette Hartmann, Max Borsche, Andreas Traschütz, Dominik Straumann, Michael Strupp, Alexander Andrea Tarnutzer

**Affiliations:** 1 Neurology, Cantonal Hospital of Baden, Baden, 5404 Switzerland; 2 Faculty of Medicine, University of Zurich, Zurich, Switzerland; 3 Department of Neurology and German Center for Vertigo and Balance Disorders, University Hospital, Ludwig-Maximilians University, Munich, Germany; 4 Department of Psychiatry and Psychotherapy, Comprehensive Center for Clinical Neurosciences and Mental Health (C3NMH), Medical University of Vienna, Vienna, Austria; 5 Institute of Neurogenetics, University of Lübeck and University Hospital Schleswig- Holstein, Campus Lübeck, Lübeck, Germany; 6 Department of Neurology, University of Lübeck and University Hospital Schleswig- Holstein, Campus Lübeck, Lübeck, Germany; 7 Research Division “Translational Genomics of Neurodegenerative Diseases”, Hertie- Institute for Clinical Brain Research and Center of Neurology, University of Tübingen, Tübingen, Germany; 8 German Center for Neurodegenerative Diseases (DZNE), University of Tübingen, Tübingen, Germany; 9 Department of Neurology, University Hospital Zurich, Zurich, Switzerland

**Keywords:** Vestibulo-ocular reflex, Eye movement recordings, Hereditary ataxia, Systematic review, Cerebellum, RFC1

## Abstract

Cerebellar ataxia, neuropathy, vestibular-areflexia syndrome (CANVAS) has been linked to biallelic intronic repeat-expansions in *RFC1*. Video-head-impulse testing (vHIT) offers a quantitative assessment of the angular vestibulo-ocular reflex (aVOR) of all three canals. We evaluated patterns of peripheral-vestibular impairment, its change over time and evaluated correlations with other parameters. PubMed/Embase were searched for articles reporting vHIT in patients with CANVAS/RFC1-related ataxia. A multiple linear-regression model was used to analyse relationships between vHIT-gains and clinical parameters (age, disease duration, sex, biallelic RFC1 expansion). A special focus was put on sequential vHIT in individual patients. 23/64 studies met inclusion criteria; additional 13 studies were identified through reference screening. Twenty-five studies reported individual vHIT-gains and demographic data, suitable for quantitative analysis. Substantial aVOR-gain reductions were found for horizontal (0.32 ± 0.02, *n* = 146 patients), anterior (0.39 ± 0.03, *n* = 27) and posterior (0.29 ± 0.03, *n* = 27) canals. Linear regression showed an association between horizontal vHIT-gains (*n* = 146 patients; range of gain: 0-1.3) and disease duration (range: 0–444 months, coef. =-0.0048, *p* = 0.031) and male sex (coef. =-0.1604, *p* < 0.001). A decline in horizontal-canal vHIT-gains at least one side over time was noted in 15/21 patients after a mean follow-up time of 33.4 ± 10.7 months. vHIT is a potential biomarker for monitoring progression of CANVAS/RFC1-related ataxia. The significant association between reduced vHIT-gains and disease duration, and their intra-individual decline over time emphasize that impairment of the aVOR reflects the underlying neurodegenerative disease process. Multi-centre prospective studies are needed for systematic early screening and longitudinal validation as outcome for future targeted therapy trials.

**Supplementary Information:**

The online version contains supplementary material available at 10.1007/s12311-025-01825-y.

## Introduction

Cerebellar ataxia with neuropathy and vestibular areflexia syndrome (CANVAS) is a slowly progressive adult-onset combined vestibular (peripheral and central) and cerebellar disorder in combination with a ganglionopathy [1]. Its main features involve gait ataxia, sensory axonal neuronopathy (ganglionopathy), bilateral vestibulopathy, chronic cough, and autonomic dysfunction [1, 2]. The clinical spectrum of this condition is broad and the onset of the different signs and symptoms varies over decades. The genetic basis of CANVAS has been identified, linking these patients to biallelic intronic repeat expansions in the *replication factor complex subunit 1* (RFC1) gene to the disease [3]. The prevalence of RFC1-related ataxia has been studied broadly, and currently it is assumed that more than 20% of all hereditary ataxias could be linked to RFC1 [4]. Furthermore, biallelic repeat expansions have been found in more than 90% of patients presenting with the phenotypic trias of CANVAS [5]. However, it must be noted that not all patients with a CANVAS phenotype have repeat expansions (or rare truncating mutations) in the RFC1 gene and not all patients demonstrate the full spectrum of RFC1-related ataxia [6]. Recently, the presence of a GAA repeat expansion in *FGF14* has also been identified among patients with a CANVAS-like phenotype [7]. Previously proposed diagnostic criteria for clinically definitive CANVAS (before the discovery of the RFC1 gene mutation) included the following: abnormal visually enhanced vestibulo-ocular reflex (VVOR); cerebellar atrophy on MRI, mainly involving vermian lobules VI, VIIa and VII; neurophysiological evidence of a sensory neuronopathy and exclusion of genetic ataxias able to be genetically tested [1]. Although, the pathology of this syndrome is not fully understood, evidence suggests that the vestibular areflexia and sensory impairment are caused by a neuronopathy (ganglionopathy) affecting both the Scarpa’s ganglia (leading to vestibular impairment) and the dorsal root ganglia (resulting in somatosensory deficits) [8]. CANVAS is relatively rare, still underdiagnosed with no specific treatment yet [9]. Thus, a better understanding of the spectrum of phenotypes could help in earlier diagnosis and improved tailoring of therapy.

The impairment of peripheral-vestibular function can be assessed quantitatively by video-based head-impulse testing (vHIT), thus providing detailed information on an impairment of the angular vestibulo-ocular reflex (aVOR) of single semi-circular canals (SCCs) [10, 11]. Other, more traditional ways to quantify the integrity of the aVOR include caloric testing and rotatory chair testing. However, they only provide information about the functional state of the horizontal SCCs [12].

While the evidence on the use of vHIT in CANVAS / RFC1-related ataxia is growing, patient numbers per study are often small and a more detailed characterization of vHIT patterns in CANVAS / RFC1-related ataxia is missing [13]. The primary aim of this systematic review and meta-analysis was to report on the prevalence and diagnostic yield of vHIT abnormalities in patients diagnosed with CANVAS / RFC1-related ataxia, focusing both on the horizontal and the vertical SCCs. We also investigated the relationship between vHIT-patterns and disease duration, age at examination and sex to better understand the temporal dynamics of aVOR-impairment. We hypothesized that most patients with CANVAS / RFC1-related ataxia will demonstrate significantly reduced aVOR-gains at the time of initial testing with further deterioration over time.

## Methods

### Search Sources and Strategy

This systematic review followed the recommendations of the Preferred Reporting Items for Systematic Review and Meta-Analyses (PRISMA) method [14]. An online database search was performed using PubMed and Embase and included both English-language articles and non-English language articles. The search strategy involved the following description: ((Cerebellar ataxia with bilateral vestibulopathy) OR (CABV) OR (CANVAS) OR (cerebellar ataxia with neuropathy and vestibular areflexia syndrome) OR (RFC-1) OR (RFC1)) AND ((Video Head Impulse) OR (vHIT) OR (quantitative vestibular)). A manual search was performed on the references of the selected articles. Studies reporting on preliminary data were included in the qualitative analysis, but not in the quantitative analysis (meta-analysis). We contacted corresponding authors where necessary. Our search was updated through August 7th, 2024. Being a systematic review of the literature and a meta-analysis, no ethical approval was necessary. No registration at PROSPERO was made.

### Study Selection and Quality Assessment

Inclusion criteria for this study were original data on human subjects with a clinically based diagnosis of CANVAS or genetically confirmed RFC1-related ataxia reporting on quantitative assessment of the aVOR by means of vHIT. Two authors (AT and MSH) independently screened all abstracts against the pre-defined inclusion criteria using a structured process. Publications selected based on the screening criteria, underwent full-text review for assessment against the pre-defined inclusion criteria to identify the final studies for the data extraction stage. Disagreements were resolved by cross-checking and discussion. Publications that did not match the inclusion criteria or did not report original data as well as conference abstracts and duplicates were excluded. We calculated interrater agreement on full-text inclusion using Cohen’s kappa [15] We assessed the risk of bias for all studies using the Downs and Black quality assessment checklist and rated the overall quality of the study (excellent, good, fair, poor) [16]. As our systematic review did not include randomized controlled trials (RCTs), the checklist was modified and questions addressing criteria related to RCTs (questions 4, 5, 8, 13–15, 17, 19, 21–25, 27) were discarded. Thus, the scores for calculating overall quality of the study were adjusted accordingly.

### Data Extraction, Synthesis and Analysis

The extracted data included individual subject vHIT-gain values for horizontal and– if provided– also for anterior and posterior semi-circular canals. For the purpose of the statistical analysis, negative aVOR gain values were set to zero. Furthermore, we also retrieved information on eye movement recording devices used and demographics on the presence of the RFC1 repeat expansions, age, disease duration and sex.

### Data Analysis

Data from the selected studies were analysed in Microsoft Excel (Version 2021) and Python (Version 3.10). A multivariate linear regression model was used to analyse the linear relationship between the vHIT values of the horizontal, anterior and posterior canal (dependent variables) versus age, disease duration, sex and the presence of RFC1 expansions (independent variables). These regressions were done by statsmodels Python package (Version 0.14.4).

Pearson correlation for the right and left ear vHIT-gain values was done using Python SciPy package (Version 1.11). The hypothesis test for its non-correlation (H0: Pearson correlation coefficient is equal to zero) was done using the scipy.stats.pearsonr function.

### Data Availability

Source data used for meta-analysis will be made available to others upon request to the corresponding author.

## Results

**Fig. 1:**
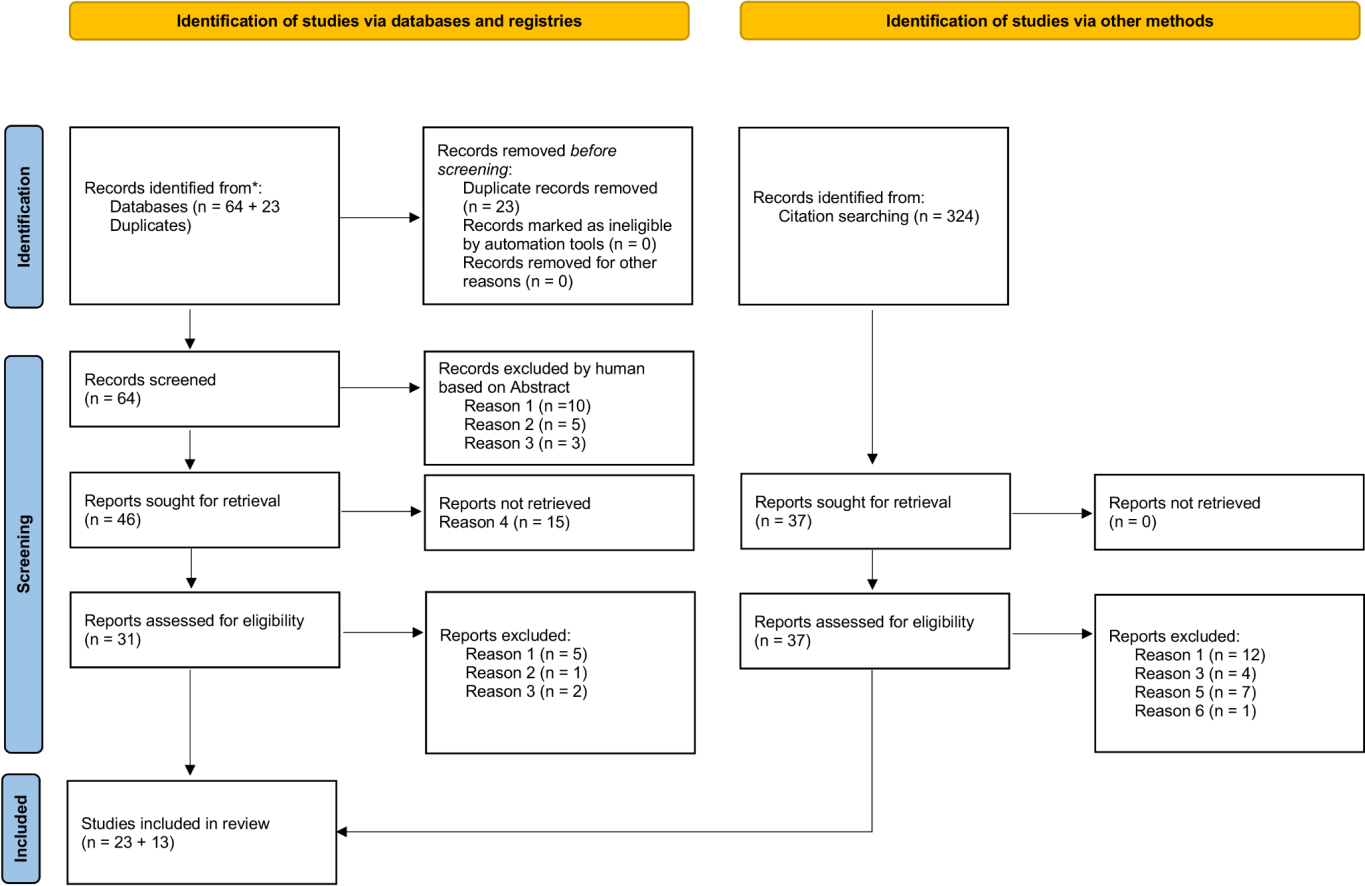
PRISMA flow chart. Studies selected for the qualitative analysis. Reasons for exclusion: Reason 1 - No data on human subjects with CANVAS or RFC1-related ataxia; Reason 2 - No original data reported; Reason 3- Not reporting on the assessment of the vestibulo-ocular reflex; Reason 4– All these publications were conference abstracts; Reason 5– Not reporting on quantitative HIT measurements; Reason 6– Publication already included

Our search conducted on August 7th 2024 identified 64 unique citations, of which 19 (29.7%) were excluded at the abstract level. Our independent raters had 65% initial agreement on inclusion of full-text manuscripts (kappa value 0.47). After resolving initial disagreements, 23/64 studies were considered eligible (Fig. 1– PRISMA flow chart), representing 35.9% of the total. An additional, manual citation search of all included manuscripts identified 324 unique citations, of which 311 (96%) were excluded at the abstract level. After resolving initial disagreements and manually removing duplicates, 13/323 studies from the manual search were considered eligible (Fig. 1– PRISMA flow chart), representing 4% of the total. Our independent raters had 98% initial agreement on inclusion of full-text manuscripts (kappa value 0.96). A total of 36 studies were included in the qualitative analysis. Full study details are provided in Supplementary Table 1 in Appendix 1.

Their publication dates ranged from 2013 to 2024. Six studies had a prospective study design and 30 were retrospective. Five were prospective case-control studies, one was a prospective cross-sectional study, 6 were retrospective observational studies, 2 were retrospective cross-sectional, 1 was a retrospective cohort study, 4 were retrospective chart reviews, 6 were case series, 6 were case reports, 4 were letters to the editor and one was a teaching video. Most of the 36 studies were performed in Germany (*n* = 7) and Italy (*n* = 7), five studies in Spain, three in Switzerland, three in Japan, two in France, two in New Zealand, followed by one in Czech Republic, Netherlands, Australia, USA, Argentina, United Kingdom and Israel. A total of 2008 patients were included in these studies, including 385 CANVAS or RFC1 positive patients. Three studies were excluded from the final analysis due to duplicates of published patients’ data ([17–19]) and seven due to non-retrievable single subject data ([20–25]). A total of 164 patients from 36 manuscripts with individual vHIT data were included in the qualitative analysis. See Appendix 1 for details on included studies. Seven studies recorded vHIT with the EyeSeeCam system (Interacoustics, Denmark), 21 used the ICS Impulse device (Natus, USA) and one used E(ye) BRAIN tracker (EyeBrain, France). Seven studies did not report on the recording device used for vHIT.

### Selected Study Characteristics

The qualitative analysis of 36 studies identified 25 studies with individual vHIT data and demographic data, suitable for a quantitative analysis. The quantitative analysis included data from 146 unique patients (54 CANVAS patients; 92 genetically confirmed RFC1-related ataxia patients). Among 54 patients with the clinical diagnosis of CANVAS 2 underwent genetic testing and did not have the pathological RFC1 expansions [26]. The genetic information on the remaining 52 patients was unknown. 83 out of 92 patients with genetically confirmed RFC1-related ataxia had a complete CANVAS diagnosis [2, 27–36]. Seventy-two females and 74 males were included in the quantitative analysis. The mean age (± standard error of the mean [SEM]) of the participants at the first vHIT examination was 67.8 ± 0.85 years. The mean disease duration (± standard error of the mean [SEM]) was 8.9 ± 0.60 years.

### Statistical Analysis of Studies Selected for Quantitative Analysis

The mean vHIT values (left and right side averaged) among patients with genetically confirmed RFC1 gene expansion were 0.31 ± 0.02 for the horizontal canal, 0.41 ± 0.05 for the anterior canal and 0.29 ± 0.04 for the posterior canal. Among patients with a clinical diagnosis of CANVAS, the mean horizontal vHIT gain was 0.34 ± 0.03, for the anterior canal 0.37 ± 0.05, and for the posterior canal 0.28 ± 0.03.

**Fig. 2:**
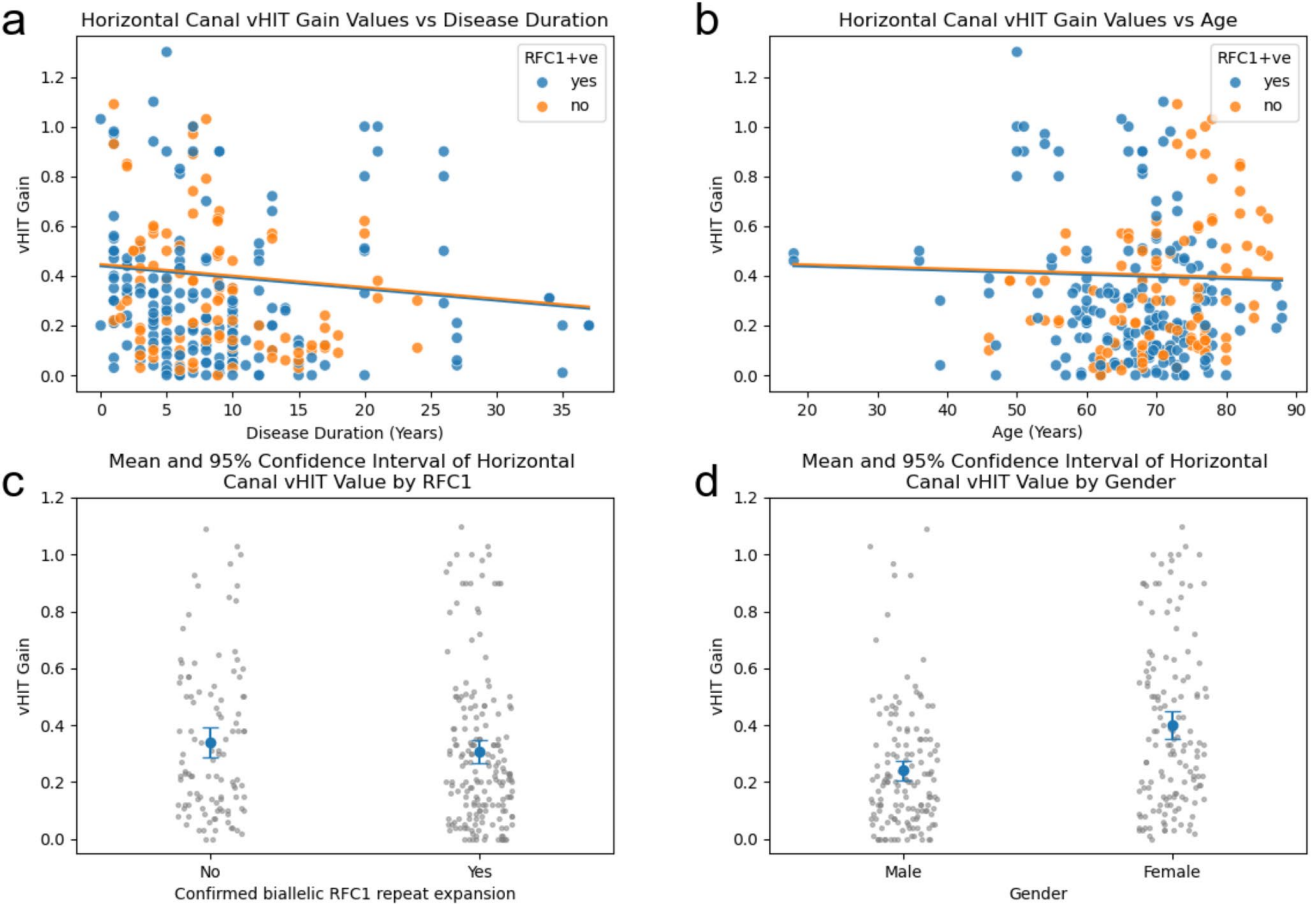
Horizontal canal characteristics. (**a**) Scatter Plot between the vHIT gain and disease duration among 146 Patients with CANVAS diagnosis or a positive RFC1 gene (coef. -0.0046, *p* = 0.035). (**b**) Scatter Plot between the vHIT gain and age at examination among 146 Patients with CANVAS diagnosis or a positive RFC1 gene (coef. -0.0008, *p* = 0.587). (**c**) A mean and 95% confidence interval of the vHIT gain value for patients with positive RFC1 gene (mean; standard error = 0.308 ± 0.020) and patients with no RFC gene or those who did not undergo genetic testing (mean; standard error = 0.340 ± 0.026). (**d**) A mean and 95% confidence interval of the vHIT gain value and the 95% Confidence intervals for males (mean = 0.241 ± 0.018) and females (mean = 0.401 ± 0.025)

A multiple linear regression model was used to analyse the relationship between the vHIT values of the horizontal, anterior and posterior canal amongst 146 patients and covariates of age at examination, disease duration, sex and the presence of the pathological RFC1 expansion for patients who had undergone genetic testing. The model confirmed a significant association between the horizontal vHIT-gain values amongst 146 patients and the disease duration (coef.=-0.0046, *p* = 0.035) (Fig. 2A) and amongst males (coef.=-0.1595, *p* < 0.001) compared to females (Fig. 2D). There was no statistically significant association between the horizontal vHIT-gain values and the tested presence of the biallelic RFC1 repeat expansion (coef.= -0.0076, *p* = 0.817) or age (coef.=- 0.0008, *p* = 0.587). These findings are illustrated in Fig. 2b and c.

As the right and left ear vHIT-gain values were positively correlated (Pearson Correlation 0.868, *p* < 0.001), we also conducted a separate linear regression for each ear. The right horizontal vHIT gains were also associated with the disease duration (coef.= -0.0067, *p* = 0.034) and male sex (coef. -0.1668, *p* < 0.001). However, the left horizontal vHIT gains showed only a significant relationship with the male sex (coef.=-0.1522, *p* = 0.001) but not with disease duration (coef.= -0.0026, *p* = 0.407), as illustrated in Appendix 2.

Data on anterior and posterior canal vHIT-gain values was available from 27 patients from 9 studies [25, 29–31, 34, 36–39]. A linear regression model showed a marginally statistically significant association between the anterior vHIT-gain values and disease duration (coef.= -0.0084, *p* = 0.050). There was no statistically significant association between anterior vHIT-gain values and age (coef.= 0.0016, *p* = 0.62), tested presence of RFC1 repeat expansions (coef.= 0.1184, *p* = 0.134) or male sex (coef.= -0.0565, *p* = 0.404), as shown in Appendix 2. For the posterior canal, there was a statistically significant relationship between the vHIT-gain value and disease duration (coef.= -0.0075, *p* = 0.025), but not for age (coef.=- 0.0007, *p* = 0.282) or the presence of the RFC1 repeat expansions (coef.= 0.0714, *p* = 0.241) or male sex (coef.= -0.0659, *p* = 0.212), as illustrated in Appendix 2. A separate linear regression of right and left anterior and posterior canals also showed no significant results (see Appendix 2 for details).

**Fig. 3:**
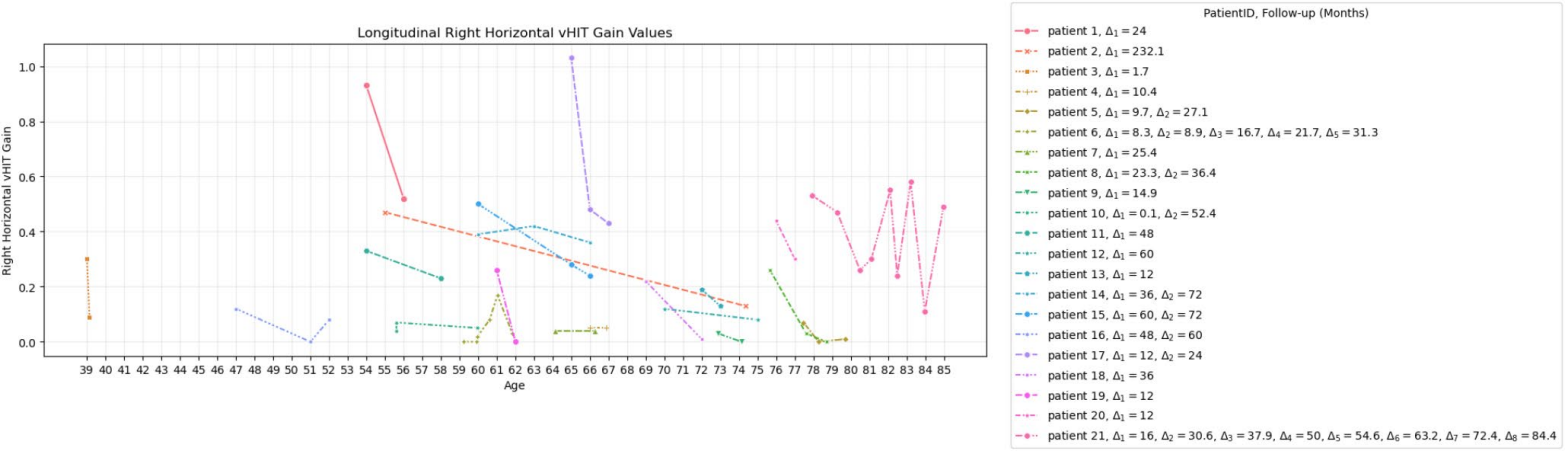
Longitudinal vHIT results for right horizontal canal Longitudinal vHIT gain values of right horizontal canal of 21 Patients and time (Δ_1−8_) between the consecutive follow-ups and first appointment

**Fig. 4:**
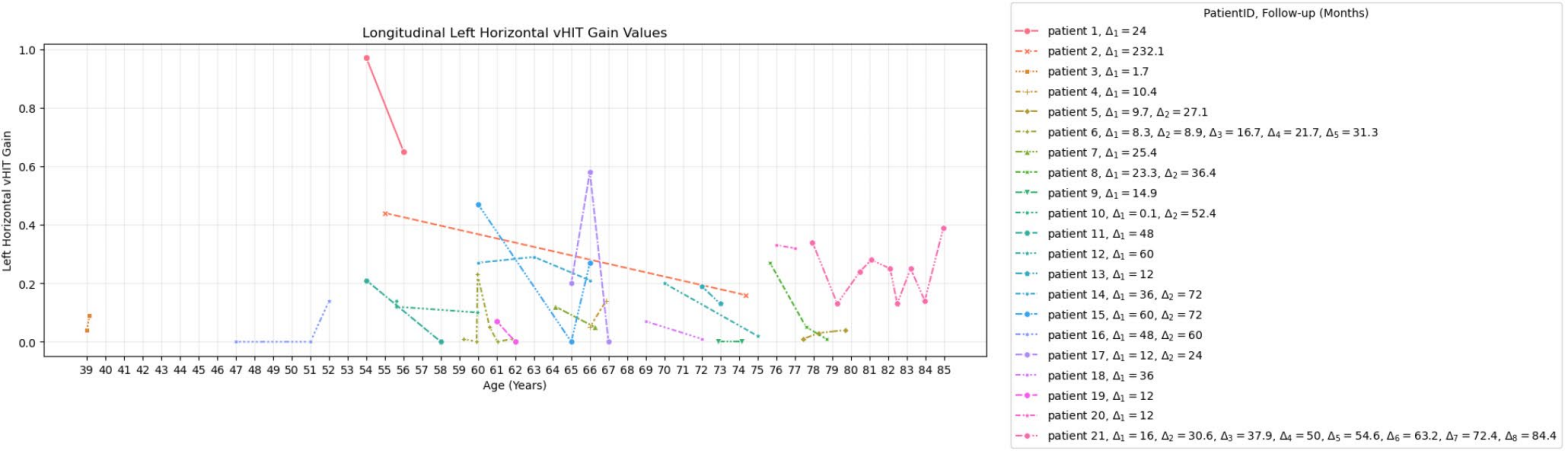
Longitudinal vHIT results for left horizontal canal Longitudinal vHIT gain values of left horizontal canal of 21 Patients and time (Δ_1−8_) between the consecutive follow-ups and first appointment

Longitudinal data on vHIT was available from 21 patients included in this analysis from five studies [6, 7, 13, 25, 40]. All these patients underwent genetic testing and were positive for a repeat-expansion in the RFC1-gene. The mean vHIT at first examination was 0.30 ± 0.06 for the right horizontal canal and 0.21 ± 0.05 for the left horizontal canal. At the first follow-up, the mean vHIT for the right horizontal canal among the 21 patients was 0.16 ± 0.04 and for the left horizontal canal, 0.13 ± 0.04. Nine of the 21 patients underwent a second follow-up appointment, with a mean vHIT gain value of 0.16 ± 0.05 for the right horizontal canal and 0.14 ± 0.03 for the left. The mean time for the first follow-up was 33.4 ± 10.7 months, and for the second follow-up, 67.9 ± 3.3 months since the first examination. Additional longitudinal data was available on patient 6 and patient 21, who had a total of 5 and 8 follow-ups, respectively. The longitudinal right horizontal vHIT gain values of 21 patients are shown in Fig. 3. Fifteen out of 21 patients demonstrated a decline in the right vHIT gain at the first follow-up. During a second follow-up, a further decline in the right vHIT gains, compared to the first examination, was observed among 5 patients (Patients 8, 14, 15, 17 and 21). Patients 5 and 8 demonstrated a slight improvement in right vHIT gains. The longitudinal left horizontal vHIT gain values are shown in Fig. 4. Fourteen out of 21 patients demonstrated a decline of the left vHIT gain at the first follow-up. During a second follow-up, a further decline in the left vHIT gains, compared to the first examination, was observed among 4 patients (Patients 8,10,14 and 17). Patients 5 and 6 demonstrated a slight improvement in left vHIT gains. Overall, looking at the first examination and the last follow-up data available, 19 out of 21 patients demonstrated a decline of the vHIT gain on at least one side over time.

## Discussion

The video head impulse test (vHIT) is a widely used tool for clinical assessment of the dizzy patient [41]. It became a universal method of a detailed, quantitative assessment of all six semicircular canals [42]. For neurodegenerative disorders such as CANVAS/RFC1-related ataxia, the ability to detect aVOR-deficits at early stages of vestibulopathy could allow for earlier disease detection and monitoring of progression.

This study aimed to systematically review the literature on the vHIT amongst patients with a clinical diagnosis of CANVAS or with a genetically confirmed repeat expansion in the RFC1 gene. The quantitative analysis involved data from a total of 146 patients (72 females and 74 males). Fifty-four of them had a clinical diagnosis of CANVAS and 92 were genetically confirmed RFC1-related ataxia patients, with a mean age of 67.8 ± 0.85 years and a mean disease of 8.9 ± 0.60 years. Overall, substantial aVOR-gain reductions were found with average gain-values below 0.40 for all canals, confirming previous observations and the hypothesis outlined at the beginning of our manuscript. Using a linear regression model, a statistically significant association between the decline in the horizontal vHIT-gain value and disease duration and male sex was demonstrated. There was no difference in vHIT gains between CANVAS patients with and without genetic test results for RFC1 repeat expansions. Furthermore, there was a significant association between the posterior canal vHIT gain values and the disease duration, and a trend for the anterior canal, where the p-value was equal to 0.05. There was no significant association between the anterior or posterior canal and age, presence of RFC1 repeat expansions or sex. However, it must be noted that the data on the anterior and posterior canal vHIT values was only available for 25 patients, thus these results may be underpowered. For CANVAS / RFC1-related ataxia, quantitative data on other oculomotor biomarkers is scarce. Presence of spontaneous nystagmus has been assessed quantitatively as well, demonstrating downbeat nystagmus in 45% of cases in one study [26]. Noteworthy, no quantitative data on other oculomotor domains such as pursuit eye movements or saccades has been published, indicating a gap of knowledge as emphasized by our group [43]. Thus, vHIT is the preferred cross-sectional and also longitudinal quantitative oculomotor biomarker in CANVAS/RFC1-related ataxia.

### The Impact of Disease Duration and Observations from Longitudinal Data

Our findings suggest that the extent of the impairment of vestibular function is related to the disease duration. The progressive neurodegeneration in CANVAS was hypothesized earlier by demonstrating that this disease involves a ganglionopathy in the central nervous system as well as in the peripheral nervous system [44]. Although the pathophysiology of CANVAS/RFC1-related ataxia is not fully understood, evidence from post-mortem studies suggest that it is caused by a neuronopathy (ganglionopathy) that affects the Scarpa’s ganglion (but spares the spiral ganglion [30]) and the dorsal root ganglion [8]. In CANVAS, the Scarpa ganglionopathy (vestibular neuropathy) leads to bilateral vestibular hypofunction and sparing of the spiral ganglion explains the normal function of the cochlear nerve. Since the vestibular system involves both peripheral and central components, progressive degeneration would affect the aVOR function, leading to decline in vHIT gains. The association between the disease duration and the decline in the vHIT gain, suggesting a progressive neurodegeneration, is further supported by our longitudinal data of 21 patients, where 18 demonstrated a decline in vHIT gains over time for at least one horizontal semicircular canal, confirming the initially outlined hypothesis at the beginning of our manuscript. The progressive course of the disease is further supported by a large retrospective study on 392 RFC1 positive patients which showed that 54% of them needed a walking aid after a median disease duration of 10 years (IQR = 5–16) and 17% required a wheelchair after 14 years (IQR *=* 11–21) [45]. Longitudinal data available for 205 cases demonstrated that after a median interval of 4 years (IQR = 2–8) from the first examination and of 12 years (IQR = 8–20) from disease onset, an extra 12% and 17% of patients respectively, developed signs of cerebellar and vestibular involvement. Overall, taking into account the data from the first follow-up and second follow-up, when possible, 195 (50%) developed complete CANVAS, compared to 122 (31%) at disease onset [45].

There was no significant association between the age at examination and the vHIT gains. We noted a large spread of age at testing, ranging from 18 years to 88 years. The mean disease duration was 8.9 ± 0.6 years. A significant number of patients (*n* = 16) had a non-pathological vHIT gain in at least one ear (12 patients bilaterally) at first examination (considering a mean gain < 0.7 as pathologic) [46, 47]. It has to be noted that 9 patients with a genetically confirmed repeat expansion in the RFC1 gene in this study, did not necessarily fulfilled all diagnostic criteria for clinical CANVAS diagnosis, one of which involves abnormal VVOR [13, 26]. In CANVAS there is also a variability in age at onset and in the speed of progression. The onset of symptoms in CANVAS starts usually around the age 50 to 60 with a relatively broad range of 19 to 82 years of age [2, 3, 48, 49]. The disease is known to have a slowly progressive course; however, it affects patients differently and unequally [48, 49]. There is also a heterogeneity in symptoms presentation. A sensory as well as autonomic dysfunction often precede the vestibular and cerebellar manifestations [2, 3, 48]. A retrospective study found that dysarthria and/or dysphagia, suggestive of cerebellar involvement, and oscillopsia, secondary to bilateral vestibular impairment or cerebellar loss of function, were less frequent in the early course of the disease [45].

### Presence of a Repeat Expansion in the RFC1 Gene

A retrospective multicentre study that measured the size of the RFC1 repeat expansion in 392 cases and using a quasi-Poisson regression model, found that a larger repeat size of both the smaller and larger allele was associated with an earlier age of onset, higher risk of developing disabling symptoms, such as dysarthria or loss of independent walking earlier in disease course [45]. This study also found that participants with larger RFC1 expansions were likely to have more complex disease phenotypes [45]. In our study compared the vHIT results among the patients with genetically confirmed repeat expansion in the RFC1 gene with those with a clinical diagnosis of CANVAS, but without genetic test results. However, we did not find an association between the presence of biallelic repeat expansions in the RFC1 gene and vHIT values. This is most likely due to missing data on the genetic testing of patients with clinical diagnosis of CANVAS. In this study only 2 patients with complete clinical diagnosis of CANVAS were known to test negative for RFC1 repeat expansions [26, 49]. The biallelic pentanucleotide repeat expansion in the gene encoding *RFC1* and its link to CANVAS was first reported in 2019 and 52 patients from 11 papers ([2, 27–36]) included in our study did not contain information on the presence of this genetic alternation and were classified as “clinical diagnosis of CANVAS” [3]. Furthermore, studies suggest that the biallelic expansion in RFC1 is identified among 82-97% of patients with full clinical diagnosis of CANVAS [3, 50]. Thus, one would expect no difference between CANVAS patients with biallelic RFC1 repeat expansions and those, who did not undergo genetic testing. This potential homogeneity between the two sample groups could provide further explanation of our findings. Other factors such as the environment can also influence the gene expression as well as disease severity. Other genes might modulate the impact of the repeat expansion in the RFC1 gene, resulting in differences in disease severity [51].

### Differences in vHIT Between Males and Females

CANVAS is equally diagnosed in males and females [52]. Our findings suggest that males with genetically confirmed repeat expansion in the RFC1 gene or clinical diagnosis of CANVAS had significantly lower vHIT gain values compared to females. The reason for lower vHIT results amongst males is unknown. Variations in neurodegenerative disease manifestations between males and females are not fully understood. A review described how clinical differences between males and females in MS, Parkinson’s disease, Alzheimer’s disease, amyotrophic lateral sclerosis and spinal muscular atrophy are likely to be linked to differences in immune mechanisms driving the neuroinflammation [53]. Possible factors that could influence the sex-differences in vHIT may include hormonal differences such as neuroprotective effects of oestrogen [54]. Animal studies on cerebral ischaemia suggest that oestrogen not only has a direct neuroprotective effect, but also indirectly affects the function of astrocytes and microglia and may also influence neuroprotection by regulating gene expression [55–59]. There is also growing evidence in its neuroprotective effect in neurodegenerative diseases such as Parkinson’s and Alzheimer’s disease. Studies have demonstrated that oestrogen is neuroprotective in 1-methyl-4-phenyl-1,2,3,6-tetrahydropyridine (MPTP)-induced nigrostriatal lesions, an animal model of idiopathic PD [60, 61]. Other reasons for lower vHIT gain values among males may include epigenetic and environmental factors. Sexual dimorphism during aging secondary to differences in male and female brain cells has been described [62].

### Limitations

Limitations of our study include the relatively small sample size, particularly regarding the data on the anterior and posterior semicircular canals as well as the limited longitudinal data with a variable follow-up time. There was substantial heterogeneity of the study design (for example in defining disease onset or the choice of vHIT recording device) and measures of reported outcomes of the studies included. Whereas the difference in the gain values of the horizontal semicircular canals across different recording devices is of minor significance, this is not necessarily the case for the vertical canals. Studies have shown that gain values for the vertical canals tend to provide higher mean values when measured with EyeSeeCam compared to ICS Impulse device [63, 64]. In our study, apart from 5 patients all vertical canals (*n* = 45) were measured with the ICS Impulse device. In those studies reporting on longitudinal changes in aVOR gains as assessed by vHIT in CANVAS /RFC-1 related ataxia patients varying ranges of peak head velocities applied by the head impulses may have affected resulting aVOR gains. However, this has not been assessed by those studies and it is unlikely that systematically higher / lower head-impulse peak velocities were applied at follow-up.

Furthermore, the lack of information on genetic testing outcomes amongst 52 of the patients with a clinical diagnosis of CANVAS could have also influenced our results. Therefore, due to a missing information on the genotype, it is challenging to reach firm conclusions regarding the severity of vestibulopathy among patients with a presence of the biallelic pentanucleotide repeat expansion in the RFC1 gene compared to patients with clinical diagnosis of CANVAS. Due to the nature of this systematic review and relying primarily on a retrospective data, we did not have information on ethnicity or other co-morbidities which could have affected the vHIT readings. Six patients included in this study had a negative vHIT aVOR gain values readings [5, 6, 13, 33, 65]. We report the data as extracted, but for the statistical analysis we assumed that negative values were equal to 0, as we consider these artificial and due to technical limitations.

## Conclusions

The vHIT represents a quick, objective and well-established method for diagnosing aVOR deficits which is widely used worldwide. It easily applicable in a clinical setting and allows for use as a screening-tool, particularly at early stages of CANVAS / *RFC1*-related ataxia. Our findings demonstrate that pathological vHIT values were associated with disease duration and the deficit declined over time, which reflects and supports the neurodegenerative character of the disease. Thus, vHIT can not only be used as a diagnostic tool, but potentially also for monitoring disease progression. The reason for lower vHIT gains amongst males with clinical or genetically confirmed CANVAS remains unknown and urges further investigation. Future work will need to focus on expanding to larger multi-centre prospective studies with a wide demographic base, to better understand the aetiology and course of CANVAS and focus on earlier detection and targeted, multi-disciplinary management.

## Electronic Supplementary Material

Below is the link to the electronic supplementary material.

Supplementary Material 1

## Data Availability

Source data used for meta-analysis will be made available to others upon request to the corresponding author.
