# Supplementary material for: The Spectrum of Peripheral-Vestibular Deficits and Their Change Over Time in CANVAS/RFC1-Related Ataxia Systematic Review and Meta-Analysis of Quantitative Head-Impulse Testing
Source: Cerebellum. 2025 Mar 20;24(3):67. doi: 10.1007/s12311-025-01825-y (PMC11926034; doi:10.1007/s12311-025-01825-y)
Supplement: Supplementary file 1 — Supplementary Material 1 [file 12311_2025_1825_MOESM1_ESM.docx]

| **Author, year (citation)**  **Appendix 1**  **Supplementary Table 1. Study Characteristics** | **Study location** | **Data collection (analysis)** | **Total Study population** | **Number of CANVAS or RFC1 +ve Patients in the Study** | **Number of patents included in the final analysis** | **Subjects (% females, % males)** | **Mean age of Patients (SD)** | **Paradigms recorded** | **Recording device used** | **Special comments** |
| --- | --- | --- | --- | --- | --- | --- | --- | --- | --- | --- |
| Borsche et al. 2022 [1] | monocentric, Germany | retrospective observational study | 20 | 20 | 6 | 50%, 50% | 71±6.87 | horizontal canals | Eye-SeeCam |  |
| Yacovino et al. 2019 [2] | monocentric, Argentina | retrospective chart review | 5 | 5 | 5 | 60%, 40% | 73±6.96 | all canals | EyeSeeCam |  |
| Cazzato et al. 2016 [3] | monocentric, Italy | case series | 4 | 4 | 0 | 25%, 75% | 65±17.07 | NA | Eye-SeeCam | Data not retrievable. |
| Costales et al. 2022 [4] | monocentric, Spain | retrospective cross-sectional study | 13 | 13 | 13 | 54%, 46% | 70.4±7.39 | horizontal canals | ICS Impulse |  |
| Dankova et al.2021 [5] | monocentric, Czech Republic | prospective case control study | 32 | 12 | 2 | 50%, 50% | 76.5±2.12 | all canals, | ICS impulse |  |
| Gisatulin et al. 2020 [6] | monocentric, Germany | prospective case control study | 457 | 26 | 14 | NA | 65.8±7.60 | horizontal canals | Eye-SeeCam |  |
| Halmágyi et al. 2021 [7] | monocentric, Australia | Letter to the editor | 1 | 1 | 1 | 0%, 100% | 68 | all canals, | ICS Impulse |  |
| Hermann et al. 2023 [8] | monocentric, France | prospective case control study | 24 | 12 | 10 | 33%, 67% | 67.4±5.36 | horizontal canals | ICS impulse |  |
| Kattah et al. 2018 [9] | monocentric, USA | retrospective chart review | 41 | 2 | 2 | 51%, 49% | 65.5±10.61 | all canals, | ICS Impulse |  |
| Maruta et al. 2019 [10] | monocentric, Japan | case report | 1 | 1 | 1 | 100%, 0% | 76 | horizontal canals | Eye-SeeCam |  |
| Moreno-Ajona et al. 2021 [11] | monocentric, Spain | retrospective chart review | 5 | 5 | 5 | 60%, 40% | 70.2±11.50 | all canals, | ICS impulse |  |
| Rey-Martinez et al. 2018 [12] | multi-center, Spain | prospective case control study | 35 | 5 | 5 | 60%, 40% | 70.4±12.68 | horizontal canals | ICS impulse |  |
| Strupp et al. 2023 [13] | monocentric, Germany | teaching video | 1 | 1 | 0 | NA | 60 | horizontal canals | ICS Impulse | Patient data already published in included publication. |
| Taki et al. 2018 [14] | monocentric, Japan | case report | 1 | 1 | 1 | 0%, 100% | 68 | horizontal canals | Eye-SeeCam |  |
| Tarnutzer et al. 2016 [15] | monocentric, Switzerland | retrospective chart review | 5 | 5 | 5 | 40%, 60% | 72.2±9.42 | all canalas | ICS Impulse |  |
| Tozza et al. 2021 [16] | monocentric, Italy | Letter to the editor | 1 | 1 | 1 | 0%, 100% | 54 | all canals | ICS Impulse |  |
| Traschütz et al. 2023 [17] | monocentric, Germany | retrospective cross-sectional study | 168 | 11 | 8 | 50%, 50% | 64.9±12.6 | horizontal canals | ICS Impulse |  |
| Traschütz et al. 2021 [18] | monocentric, Germany | letter to the editor | 70 | 70 | 9 | 44%, 56% | 70.4±9.74 | horizontal canals | ICS Impulse |  |
| Tozza et al. 2020 [19] | monocentric, Italy | case report | 1 | 1 | 1 | 0%, 100% | 85 | NA | ICS Impulse | Data not retrievable |
| Infante et al. 2018 [20] | monocentric, Italy | case series | 5 | 5 | 5 | 80%, 20% | 64.6±3.85 | horizontal canals | NA |  |
| Wu et al. 2014 [21] | multicentric, New Zeeland | prospective cross-sectional study | 26 | 26 | 18 | 65%, 35% | 66.2±9.13 | horizontal canals | NA | Patients 2, 15,16, 19, 21, 22, 23 abd 26 removed as missing vHIT data. |
| Nakamura et al. 2017 [22] | monocentric, Japan | case report | 1 | 1 | 1 | 100%, 0% | 82 | horizontal canals | ICS Impulse |  |
| Mila De La Roca et al. 2018 [23] | monocentric, Spain | case series | 4 | 4 | 4 | 50%, 50% | 68.8±9.43 | NA | NA | Data not retrievable |
| Rust et al. 2017 [24] | monocentric, Switzerland | letter to the editor | 1 | 1 | 1 | 0%, 100% | 65 | all canals, | ICS Impulse |  |
| Ahmad et al. 2018 [25] | monocentric, United Kingdom | case report | 3 | 3 | 0 | 67%, 33% | 76.7±2.31 | NA | NA | Patient already published in included publication. |
| Beecroft et al. 2020 [26] | monocentric, New Zeeland | retrospective observational study | 15 | 13 | 8 | 46%, 54% | 59.4±17.3 | horizontal canals | NA | Patients M2 III:5 and M6 I:1 excluded as disease duration is unclear |
| Huin et al. 2022 [27] | monocentric, France | retrospective observational study | 50 | 50 | NA | NA | NA | NA | EyeBrain eye tracker | Data not retrievable |
| Feil et al. 2019 [28] | monocentric, Germany | retrospective observational study | 459 | 37 | 13 | 69%, 31% | 74.7±6.25 | horizontal canals, VEMPs | EyeSeeCam |  |
| Fernandez-Rueda et al. 2023 [29] | monocentric, Spain | retrospective cohort study | 7 | 7 | 7 | 86%, 14% | 59.7±10.69 | horizontal canals, VEMPs | ICS impulse |  |
| Di Rauso et.al 2023 [30] | monocentric, Italy | case series | 5 | 5 | 5 | 60%, 40% | NA | all canals | ICS impulse |  |
| Petersen et al. 2013 [31] | monocentric, Switzerland | case report | 1 | 1 | 1 | 100%, 0% | 75 | horizontal canals | ICS Impulse | Patient data already published in included publication. |
| Moyaert et al. 2023 [32] | multicentric, Netherlands | retrospective observational study | 315 | 7 | ? |  |  |  | NA | Data not retrievable |
| Azzimonti et al. 2022 [33] | monocentric, Italy | case series | 2 | 2 | NA | 0%, 100% | 41.5±13.4 | NA | NA | Data not retrievable |
| Pellerin et.al. 2024 [34] | monocentric, Germany | retrospective observational study | 203 | 17 | 4 | 50%, 50% | 67.3±8.44 | horizontal canals | ICS Impulse |  |
| Gordon et. al. 2024 [35] | monocentric, Israel | case series | 4 | 4 | 2 | 50%, 50% | 56.5±14.8 | all canals | ICS Impulse |  |
| Marcelli et al. 2024 [36] | monocentric, Italy | prospective case-control study | 22 | 6 | 6 | NA | NA | NA | ICS Impulse | Data not retrievable |

**Appendix 2**

Linear regression results for right and left horizontal canal (A), left horizontal canal only (B), right horizontal canal only (C), right and left posterior canal (D) and right and left anterior canal (E).

A

| Horizontal vHIT Value Linear Regression | | | | | | |
| --- | --- | --- | --- | --- | --- | --- |
| Dep. Variable: | vHIT_gain | R-squared: | 0.101 |  |  |  |
| Model: | OLS | Adj. R-squared: | 0.088 |  |  |  |
| Method: | Least Squares | F-statistic: | 8.044 |  |  |  |
| Date: | 19 Oct 2024 | Prob (F-statistic): | 3.69E-06 |  |  |  |
| Time: | 14:17:15 | Log-Likelihood: | -20.2 |  |  |  |
| No. Observations: | 292 | AIC: | 50.4 |  |  |  |
| Df Residuals: | 287 | BIC: | 68.78 |  |  |  |
| Df Model: | 4 |  |  |  |  |  |
| Covariance Type: | nonrobust |  |  |  |  |  |
|  | coef | std err | t | P>\|t\| | [0.025 | 0.975] |
| const | 0.5027 | 0.112 | 4.478 | 0 | 0.282 | 0.724 |
| Disease_duration | -0.0046 | 0.002 | -2.118 | 0.035 | -0.009 | 0 |
| Age | -0.0008 | 0.002 | -0.544 | 0.587 | -0.004 | 0.002 |
| RFC1_ve_yes | -0.0076 | 0.033 | -0.232 | 0.817 | -0.072 | 0.057 |
| Gender_Male | -0.1595 | 0.031 | -5.149 | 0 | -0.22 | -0.099 |
| Omnibus: | 39.119 | Durbin-Watson: | 1.634 |  |  |  |
| Prob(Omnibus): | 0 | Jarque-Bera (JB): | 50.608 |  |  |  |
| Skew: | 0.973 | Prob(JB): | 1.02E-11 |  |  |  |
| Kurtosis: | 3.61 | Cond. No. | 510 |  |  |  |

B

| Left Horizontal vHIT Value Linear Regression | | | | | | |
| --- | --- | --- | --- | --- | --- | --- |
| Dep. Variable: | vHIT_gain | R-squared: | 0.089 |  |  |  |
| Model: | OLS | Adj. R-squared: | 0.064 |  |  |  |
| Method: | Least Squares | F-statistic: | 3.458 |  |  |  |
| Date: | 19 Oct 2024 | Prob (F-statistic): | 0.00995 |  |  |  |
| Time: | 14:17:15 | Log-Likelihood: | -9.4125 |  |  |  |
| No. Observations: | 146 | AIC: | 28.82 |  |  |  |
| Df Residuals: | 141 | BIC: | 43.74 |  |  |  |
| Df Model: | 4 |  |  |  |  |  |
| Covariance Type: | nonrobust |  |  |  |  |  |
|  | coef | std err | t | P>\|t\| | [0.025 | 0.975] |
| const | 0.4715 | 0.159 | 2.957 | 0.004 | 0.156 | 0.787 |
| Disease_duration | -0.0026 | 0.003 | -0.832 | 0.407 | -0.009 | 0.004 |
| Age | -0.0006 | 0.002 | -0.266 | 0.791 | -0.005 | 0.004 |
| RFC1_ve_yes | -0.025 | 0.047 | -0.538 | 0.592 | -0.117 | 0.067 |
| Gender_Male | -0.1522 | 0.044 | -3.459 | 0.001 | -0.239 | -0.065 |
| Omnibus: | 17.134 | Durbin-Watson: | 1.651 |  |  |  |
| Prob(Omnibus): | 0 | Jarque-Bera (JB): | 19.612 |  |  |  |
| Skew: | 0.883 | Prob(JB): | 5.51E-05 |  |  |  |
| Kurtosis: | 3.325 | Cond. No. | 510 |  |  |  |

C

| Right Horizontal vHIT Value Linear Regression | | | | | | |
| --- | --- | --- | --- | --- | --- | --- |
| Dep. Variable: | vHIT_gain | R-squared: | 0.119 |  |  |  |
| Model: | OLS | Adj. R-squared: | 0.094 |  |  |  |
| Method: | Least Squares | F-statistic: | 4.777 |  |  |  |
| Date: | 19 Oct 2024 | Prob (F-statistic): | 0.00121 |  |  |  |
| Time: | 14:17:16 | Log-Likelihood: | -10.139 |  |  |  |
| No. Observations: | 146 | AIC: | 30.28 |  |  |  |
| Df Residuals: | 141 | BIC: | 45.2 |  |  |  |
| Df Model: | 4 |  |  |  |  |  |
| Covariance Type: | nonrobust |  |  |  |  |  |
|  | coef | std err | t | P>\|t\| | [0.025 | 0.975] |
| const | 0.534 | 0.16 | 3.333 | 0.001 | 0.217 | 0.851 |
| Disease_duration | -0.0067 | 0.003 | -2.139 | 0.034 | -0.013 | -0.001 |
| Age | -0.0011 | 0.002 | -0.499 | 0.619 | -0.005 | 0.003 |
| RFC1_ve_yes | 0.0098 | 0.047 | 0.21 | 0.834 | -0.083 | 0.102 |
| Gender_Male | -0.1668 | 0.044 | -3.774 | 0 | -0.254 | -0.079 |
| Omnibus: | 25.882 | Durbin-Watson: | 1.607 |  |  |  |
| Prob(Omnibus): | 0 | Jarque-Bera (JB): | 33.153 |  |  |  |
| Skew: | 1.082 | Prob(JB): | 6.32E-08 |  |  |  |
| Kurtosis: | 3.877 | Cond. No. | 510 |  |  |  |

D

| Posterior vHIT Value Linear Regression | | | | | | |
| --- | --- | --- | --- | --- | --- | --- |
| Dep. Variable: | vHIT_gain | R-squared: | 0.117 |  |  |  |
| Model: | OLS | Adj. R-squared: | 0.045 |  |  |  |
| Method: | Least Squares | F-statistic: | 1.618 |  |  |  |
| Date: | 19 Oct 2024 | Prob (F-statistic): | 0.185 |  |  |  |
| Time: | 14:17:16 | Log-Likelihood: | 17.665 |  |  |  |
| No. Observations: | 54 | AIC: | -25.33 |  |  |  |
| Df Residuals: | 49 | BIC: | -15.39 |  |  |  |
| Df Model: | 4 |  |  |  |  |  |
| Covariance Type: | nonrobust |  |  |  |  |  |
|  | coef | std err | t | P>\|t\| | [0.025 | 0.975] |
| const | 0.3041 | 0.186 | 1.639 | 0.108 | -0.069 | 0.677 |
| Disease_duration | -0.0075 | 0.003 | -2.309 | 0.025 | -0.014 | -0.001 |
| Age | 0.0007 | 0.003 | 0.282 | 0.779 | -0.004 | 0.006 |
| RFC1_ve_yes | 0.0714 | 0.06 | 1.186 | 0.241 | -0.05 | 0.192 |
| Gender_Male | -0.0659 | 0.052 | -1.265 | 0.212 | -0.171 | 0.039 |
| Omnibus: | 7.691 | Durbin-Watson: | 2.548 |  |  |  |
| Prob(Omnibus): | 0.021 | Jarque-Bera (JB): | 6.99 |  |  |  |
| Skew: | 0.854 | Prob(JB): | 0.0304 |  |  |  |
| Kurtosis: | 3.438 | Cond. No. | 524 |  |  |  |

E

| Anterior vHIT Value Linear Regression | | | | | | |
| --- | --- | --- | --- | --- | --- | --- |
| Dep. Variable: | vHIT_gain | R-squared: | 0.091 |  |  |  |
| Model: | OLS | Adj. R-squared: | 0.017 |  |  |  |
| Method: | Least Squares | F-statistic: | 1.225 |  |  |  |
| Date: | 19 Oct 2024 | Prob (F-statistic): | 0.312 |  |  |  |
| Time: | 14:17:16 | Log-Likelihood: | 3.9089 |  |  |  |
| No. Observations: | 54 | AIC: | 2.182 |  |  |  |
| Df Residuals: | 49 | BIC: | 12.13 |  |  |  |
| Df Model: | 4 |  |  |  |  |  |
| Covariance Type: | nonrobust |  |  |  |  |  |
|  | coef | std err | t | P>\|t\| | [0.025 | 0.975] |
| const | 0.3207 | 0.239 | 1.34 | 0.187 | -0.16 | 0.802 |
| Disease_duration | -0.0084 | 0.004 | -2.006 | 0.05 | -0.017 | ###### |
| Age | 0.0016 | 0.003 | 0.499 | 0.62 | -0.005 | 0.008 |
| RFC1_ve_yes | 0.1184 | 0.078 | 1.524 | 0.134 | -0.038 | 0.274 |
| Gender_Male | -0.0565 | 0.067 | -0.841 | 0.404 | -0.192 | 0.079 |
| Omnibus: | 6.456 | Durbin-Watson: | 2.543 |  |  |  |
| Prob(Omnibus): | 0.04 | Jarque-Bera (JB): | 5.51 |  |  |  |
| Skew: | 0.742 | Prob(JB): | 0.0636 |  |  |  |
| Kurtosis: | 3.495 | Cond. No. | 524 |  |  |  |

**Appendix 3**

**Fig. A1 Anterior canal characteristics.**

a. Scatter Plots showing a marginally significant correlation between the vHIT gain and disease duration (coef. -0.0084, p=0.050) among 27 Patients. b. Scatter Plot showing no correlation between the vHIT gain and age at examination (coef. 0.0016, p=0.620). c. A mean and 95% confidence interval of the vHIT gain value for patients with positive RFC1 gene (mean =0.408±0.046) and patients with no RFC gene or those who did not undergo genetic testing (mean=0.366±0.046). d. A mean and 95% confidence interval of the vHIT gain value and the 95% Confidence intervals for males (mean=0.373±0.047) and females (mean=0.403±0.045).

**
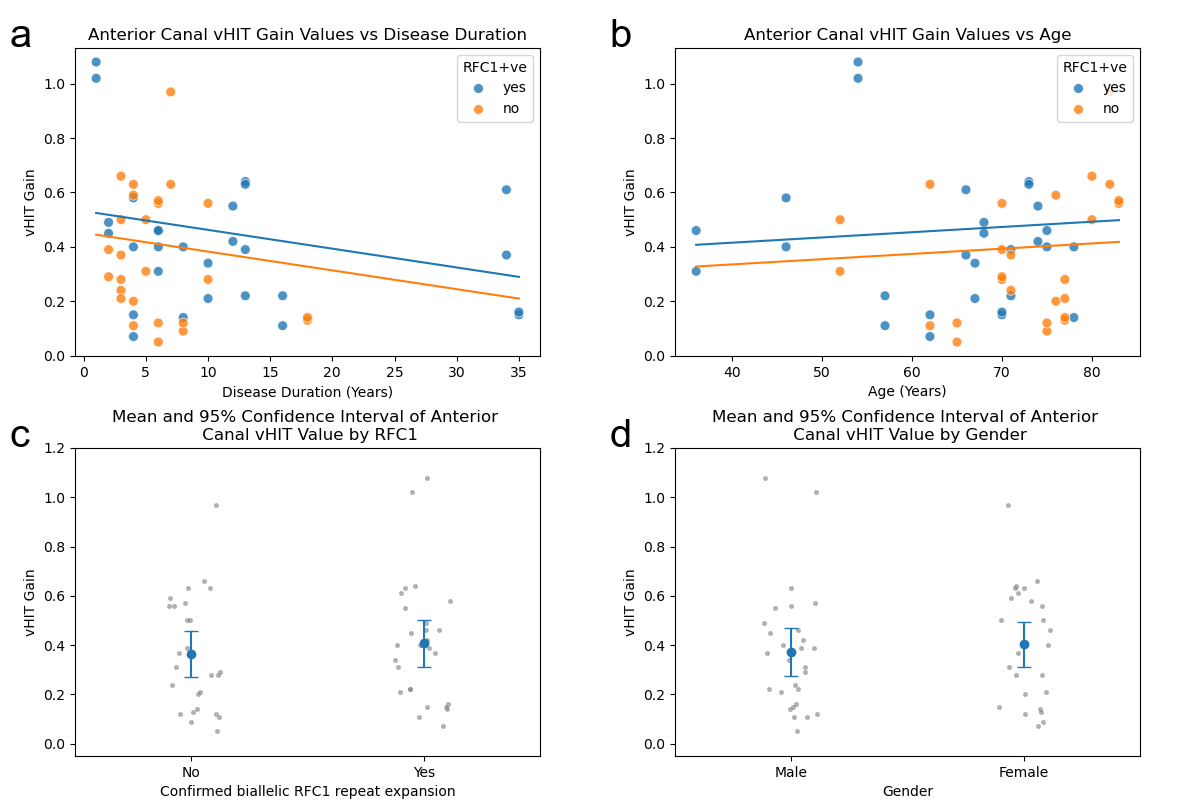
**

**
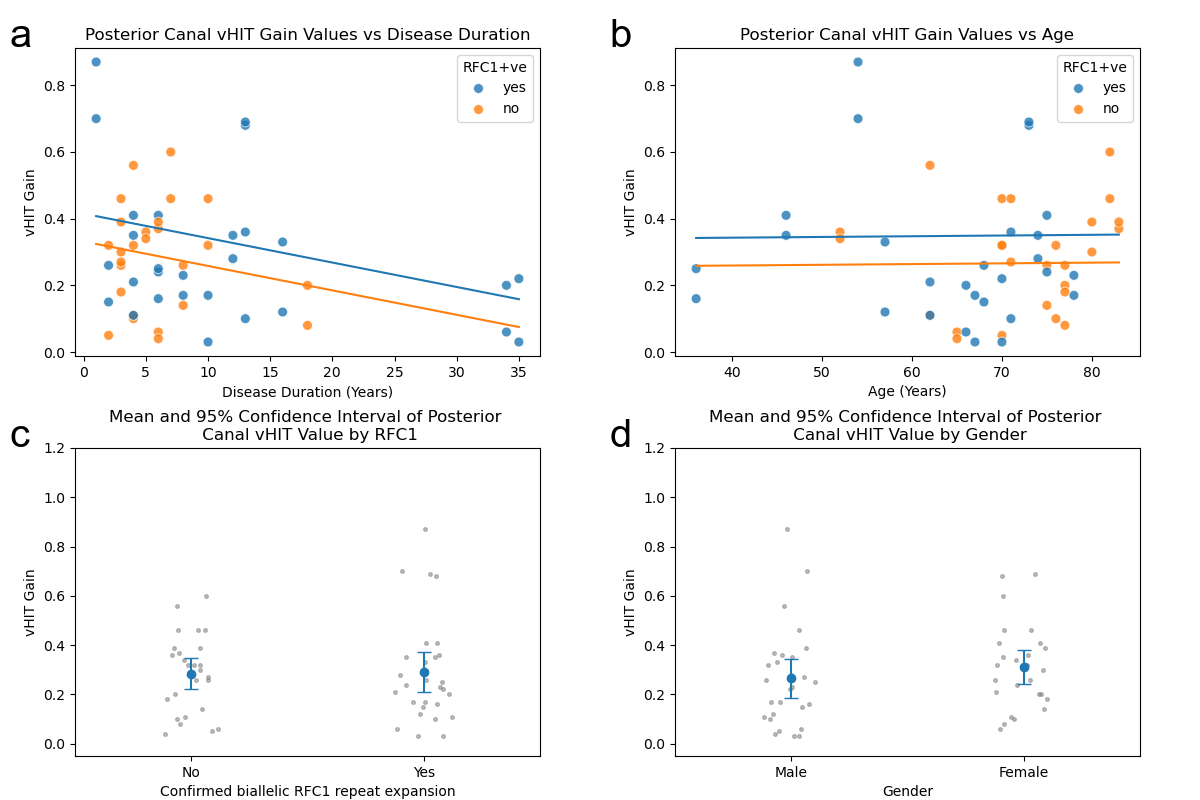
**

**Fig. A2 Posterior canal characteristics.**

a. Scatter Plots showing statistically significant correlation between the vHIT gain and disease duration (coef. -0.0075, p=0.025), among 27 Patients. b. Scatter Graph showing lack of correlation between the vHIT gain and age at examination (coef. 0.0007, p=0.779). c. A mean and 95% confidence interval of the vHIT gain value for patients with positive RFC1 gene (mean =0.291±0.040) and patients with no RFC gene or those who did not undergo genetic testing (mean=0.285±0.031). d. A mean and 95% confidence interval of the vHIT gain value and the 95% Confidence intervals for males (mean=0.265±0.038) and females (mean=0.313±0.034).

**Supplementary Data**

**Fig. A3 Right horizontal longitudinal vHIT data**

Patient number 1 (Tozza 2021 et al.) is a 54-year-old male with a vHIT result of 0.93 at first examination, 0.52 at first follow-up[16]. Patient number 2 (Traschütz 2023 et al.) is a 55-year-old male with a vHIT results of 0.47 at first examination, 0.13 at first follow up [17]. Patient number 3 (Traschütz 2023 et al.) is a 39-year-old female with a vHIT of 0.3 at first examination, 0.09 at first follow-up [17]. Patient number 4 is a 66-year-old male (Traschütz 2023 et al.) with a vHIT result of 0.05 at first examination and 0.05 at first follow-up[17]. Patient number 5 (Traschütz 2021 et al.) is a 77-year-old male with a vHIT result of 0.07 at first examination, 0.00 at first follow-up and 0.01 at second follow-up [18]. Patient number 6 (Traschütz 2021 et al.) is 59-year-old male with a vHIT test result of 0.00 at first examination, 0.00 at first follow-up and 0.02 at second follow-up, 0.08 at third follow-up, 0.17 at fourth follow-up and 0.03 at fifth follow-up[18]. Patient number 7 (Traschütz 2021 et.al.) is a 55-year-old male with vHIT result of 0.04 at first examination, 0.07 at first follow-up and 0.05 at second follow-up [18]. Patient number 8 (Traschütz 2021 et.al.) is a 75-year-old female with a vHIT result of 0.26 at first examination, 0.03 at first follow-up and 0.01 at second follow-up [18]. Patient number 9 (Traschütz 2021 et.al.) is a 72-year-old male with a vHIT result of 0.03 at first examination and 0.00 at first follow-up.[18] Patient number 10 (Traschütz 2021 et al.) is a 55-year-old male with vHIT result of 0.04 at first examination, 0.07 at first follow-up and 0.05 at second follow-up [18]. Patient number 11 (Borsche et al. 2022) is 58 years old with a vHIT result of 0.33 at first examination and 0.23 at first follow-up[1]. Patient number 12 (Borsche et al. 2022) is 70 years old with a vHIT result of 0.12 at first examination and 0.08 at first follow-up[1]. Patient number 13 (Borsche et al. 2022) is 72 years old with a vHIT result of 0.12 at first examination and 0.13 at first follow-up [1]. Patient number 14 (Borsche et al. 2022) is 60 years old with a vHIT result of 0.39 at first examination and 0.42 at first follow-up and 0.26 at second follow-up [1]. Patient number 15 (Borsche et al. 2022) is 60 years old with a vHIT result of 0.5 at first examination and 0.28 at first follow-up and 0.24 at second follow-up [1]. Patient number 16 (Borsche et al. 2022) is 47 years old with a vHIT result of 0.12 at first examination and 0.00 at first follow-up and 0.08 at second follow-up [1]. Patient number 17 (Borsche et al. 2022) is 65 years old with a vHIT result of 1.03 at first examination and 0.48 at first follow-up and 0.43 at second follow-up [1]. Patient number 18 (Borsche et al. 2022) is 69 years old with a vHIT result of 0.22 at first examination and 0.01 at first follow-up [1]. Patient number 19 (Borsche et al. 2022) is 61 years old with a vHIT result of 0.26 at first examination and 0.00 at first follow-up [1]. Patient number 20 (Borsche et al. 2022) is 76 years old with a vHIT result of 0.44 at first examination and 0.08 at first follow-up [1]. Patient number 21 (Pellerin et al. 2024) is a 77 years old female with a vHIT result of 0.53 at first examination, 0.47 at first follow-up, 0.26 at second follow-up, 0.30 at third follow-up, 0.55 at fourth follow-up, 0.24 at sixth follow up, 0.58 at seventh follow-up, 0.11 at eighth follow-up and 0.49 at ninth follow-up [34].

**Fig. A4 Left horizontal longitudinal vHIT data**

Patient number 1 (Tozza 2021 et al.) is a 54-year-old male with a vHIT result of 0.97 at first examination, 0.65 at first follow-up [16]. Patient number 2 (Traschütz 2023 et al.) is a 55-year-old male with a vHIT results of 0.44 at first examination and 0.16 at first follow up [17]. Patient number 3 (Traschütz 2023 et al.) is a 39-year-old female with a vHIT of 0.4 at first examination, 0.09 at first follow-up [17]. Patient number 4 (Traschütz 2023 et al.) is a 66-year-old male with a vHIT result of 0.05 at first examination and 0.14 at first follow-up [17]. Patient number 5 (Traschütz 2021 et al.) is a 77-year-old male with a vHIT result of 0.01 at first examination, 0.00 at first follow-up and 0.04 at second follow-up [37]. Patient number 6 (Traschütz 2021 et al.) is 59-year-old male with a vHIT test result of 0.01 at first examination, 0.00 at first follow-up and 0.23 at second follow-up [37]. Patient number 7 (Traschütz 2021 et al.) is a 64-year-old male with a vHIT result of 0.12 at first examination and 0.05 at first follow-up [37]. Patient number 8 (Traschütz 2021 et al.) is a 75-year-old female with a vHIT result of 0.27 at first examination, 0.05 at first follow-up and 0.01 at second follow-up [37]. Patient number 9 (Traschütz 2021 et al.) is a 72-year-old male with a vHIT result of 0.00 at first examination and 0.00 at first follow-up [37]. Patient number 10 (Traschütz 2021 et al.) is a 55-year-old male with vHIT result of 0.14 at first examination, 0.12 at first follow-up and 0.10 at second follow-up [37]. Patient number 11 (Borsche et al. 2022) is a 58-year-old with a vHIT result of 0.21 at first examination and 0.00 at first follow-up [1]. Patient number 12 (Borsche et al. 2022) is 70 years old with a vHIT result of 0.02 at first examination and 0.02 at first follow-up [1]. Patient number 13 (Borsche et al. 2022) is 72 years old with a vHIT result of 0.19 at first examination and 0.13 at first follow-up [1]. Patient number 14 (Borsche et al. 2022) is 60 years old with a vHIT result of 0.27 at first examination and 0.29 at first follow-up and 0.21 at second follow-up [1]. Patient number 15 (Borsche et al. 2022) is 60 years old with a vHIT result of 0.47 at first examination and 0.00 at first follow-up and 0.27 at second follow-up [1]. Patient number 16 (Borsche et al. 2022) is 47 years old with a vHIT result of 0.00 at first examination and 0.00 at first follow-up and 0.14 at second follow-up [1]. Patient number 17 (Borsche et al. 2022) is 65 years old with a vHIT result of 0.2 at first examination and 0.58 at first follow-up and -0.09 at second follow-up [1]. Patient number 18 (Borsche et al. 2022) is 69 years old with a vHIT result of 0.07 at first examination and 0.01 at first follow-up [1]. Patient number 19 (Borsche et al. 2022) is 61 years old with a vHIT result of 0.07 at first examination and 0.00 at first follow-up [1]. Patient number 20 (Borsche et al. 2022) is 76 years old with a vHIT result of 0.33 at first examination and 0.32 at first follow-up [1]. Patient number 21 (Pellerin et al.2024) is a 77 years old female with a vHIT result of 0.34 at first examination, 0.13 at first follow-up, 0.24 at second follow-up, 0.28 at third follow-up, 0.25 at fourth follow-up, 0.13 at sixth follow up, 0.25 at seventh follow-up, 0.14 at eighth follow-up and 0.39at ninth follow-up [34].

References

[1] M. Borsche *et al*, "Head impulse testing in bilateral vestibulopathy in patients with genetically defined CANVAS," *Brain and Behavior,* vol. 12, *(6),* pp. e32546, 2022.

[2] D. A. Yacovino, E. Zanotti and T. C. Hain, "Is cerebellar ataxia, neuropathy, and vestibular areflexia syndrome (CANVAS) a vestibular ganglionopathy?" *The Journal of International Advanced Otology,* vol. 15, *(2),* pp. 304, 2019.

[3] D. Cazzato *et al*, "Cerebellar ataxia, neuropathy, and vestibular areflexia syndrome: a slowly progressive disorder with stereotypical presentation," *J. Neurol.,* vol. 263, pp. 245–249, 2016.

[4] M. Costales *et al*, "CANVAS: a new genetic entity in the otorhinolaryngologist’s differential diagnosis," *Otolaryngology–Head and Neck Surgery,* vol. 166, *(1),* pp. 74–79, 2022.

[5] M. Dankova *et al*, "Clinical dynamic visual acuity in patients with cerebellar ataxia and vestibulopathy," *Plos One,* vol. 16, *(7),* pp. e0255299, 2021.

[6] M. Gisatulin *et al*, "Clinical spectrum of the pentanucleotide repeat expansion in the RFC1 gene in ataxia syndromes," *Neurology,* vol. 95, *(21),* pp. e2912–e2923, 2020.

[7] G. M. Halmágyi and D. J. Szmulewicz, "Vestibular function testing in patients with RFC1 mutations," *J. Neurol.,* vol. 268, *(12),* pp. 4894–4896, 2021.

[8] R. Hermann *et al*, "Catch-Up Saccades in Vestibular Hypofunction: A Contribution of the Cerebellum?" *The Cerebellum,* vol. 23, *(1),* pp. 136–143, 2024.

[9] J. C. Kattah, "Clinical characteristics and etiology of bilateral vestibular loss in a cohort from central Illinois," *Frontiers in Neurology,* vol. 9, pp. 336627, 2018.

[10] K. Maruta, M. Aoki and Y. Sonoda, "Cerebellar ataxia with neuropathy and vestibular areflexia syndrome (CANVAS): a case report," *Rinsho Shinkeigaku= Clinical Neurology,* vol. 59, *(1),* pp. 27–32, 2018.

[11] D. Moreno-Ajona *et al*, "VEMPs and dysautonomia assessment in definite cerebellar ataxia, neuropathy, vestibular areflexia syndrome (CANVAS): a case series study," *The Cerebellum,* vol. 20, pp. 717–723, 2021.

[12] J. Rey-Martinez *et al*, "Mathematical methods for measuring the visually enhanced vestibulo–ocular reflex and preliminary results from healthy subjects and patient groups," *Frontiers in Neurology,* vol. 9, pp. 69, 2018.

[13] M. Strupp *et al*, "Teaching video neuroImage: one bedside test, 2 clinical signs: one vestibular, the other ocular motor," *Neurology,* vol. 97, *(5),* pp. e541–e542, 2021.

[14] M. Taki *et al*, "Cerebellar ataxia with neuropathy and vestibular areflexia syndrome (CANVAS)," *Auris Nasus Larynx,* vol. 45, *(4),* pp. 866–870, 2018.

[15] A. A. Tarnutzer *et al*, "Disease-specific sparing of the anterior semicircular canals in bilateral vestibulopathy," *Clinical Neurophysiology,* vol. 127, *(8),* pp. 2791–2801, 2016.

[16] S. Tozza *et al*, "The first two-year follow-up in a patient with isolated sensory neuronopathy due to biallelic expansion in RFC1 gene," *Acta Neurol. Belg.,* vol. 123, *(4),* pp. 1601–1604, 2023.

[17] A. Traschütz *et al*, "Frequency and phenotype of RFC1 repeat expansions in bilateral vestibulopathy," *Neurology,* vol. 101, *(10),* pp. e1001–e1013, 2023.

[18] A. Traschütz *et al*, "Natural history, phenotypic spectrum, and discriminative features of multisystemic RFC1 disease," *Neurology,* vol. 96, *(9),* pp. e1369–e1382, 2021.

[19] S. Tozza *et al*, "Bedside head impulse test: a useful tool for patients with sensory ataxia," *Neurology: Genetics,* vol. 7, *(1),* pp. e541, 2020.

[20] J. Infante *et al*, "Cerebellar ataxia, neuropathy, vestibular areflexia syndrome (CANVAS) with chronic cough and preserved muscle stretch reflexes: evidence for selective sparing of afferent Ia fibres," *J. Neurol.,* vol. 265, pp. 1454–1462, 2018.

[21] T. Y. Wu *et al*, "Autonomic dysfunction is a major feature of cerebellar ataxia, neuropathy, vestibular areflexia ‘CANVAS’syndrome," *Brain,* vol. 137, *(10),* pp. 2649–2656, 2014.

[22] H. Nakamura *et al*, "Long-read sequencing identifies the pathogenic nucleotide repeat expansion in RFC1 in a Japanese case of CANVAS," *J. Hum. Genet.,* vol. 65, *(5),* pp. 475–480, 2020.

[23] A. M. M. de la Roca *et al*, "Instability in patients with CANVAS: can computerized dynamic posturography help in diagnosis?" *The Journal of International Advanced Otology,* vol. 14, *(1),* pp. 130, 2018.

[24] H. Rust *et al*, "VEMPs in a patient with cerebellar ataxia, neuropathy and vestibular areflexia (CANVAS)," *J. Neurol. Sci.,* vol. 378, pp. 9–11, 2017.

[25] H. Ahmad *et al*, "Clinical and functional characterization of a missense ELF2 variant in a CANVAS family," *Frontiers in Genetics,* vol. 9, pp. 330919, 2018.

[26] A. Cortese *et al*, "Cerebellar ataxia, neuropathy, vestibular areflexia syndrome due to RFC1 repeat expansion," *Brain,* vol. 143, *(2),* pp. 480–490, 2020.

[27] V. Huin *et al*, "Motor neuron pathology in CANVAS due to RFC1 expansions," *Brain,* vol. 145, *(6),* pp. 2121–2132, 2022.

[28] K. Feil *et al*, "What is behind cerebellar vertigo and dizziness?" *The Cerebellum,* vol. 18, pp. 320–332, 2019.

[29] M. Fernandez-Rueda, A. García-Fernández and J. J. De Vergas-Gutiérrez, "Variability in the Results of Vestibular Assessment in Patients with Genetically Confirmed Cerebellar Ataxia, Neuropathy, and Vestibular Areflexia Syndrome," *The Journal of International Advanced Otology,* vol. 19, *(5),* pp. 383, 2023.

[30] G. Di Rauso *et al*, "Speech, Gait, and Vestibular Function in Cerebellar Ataxia with Neuropathy and Vestibular Areflexia Syndrome," *Brain Sciences,* vol. 13, *(10),* pp. 1467, 2023.

[31] J. A. Petersen, W. W. Wichmann and K. P. Weber, "The pivotal sign of CANVAS," *Neurology,* vol. 81, *(18),* pp. 1642–1643, 2013.

[32] J. Moyaert *et al*, "Etiologies and hearing status in bilateral vestibulopathy: a retrospective study of 315 patients," *Frontiers in Neurology,* vol. 14, pp. 1271012, 2023.

[33] M. Azzimonti *et al*, "Association between inflammatory central nervous system lesions and Cerebellar Ataxia, Neuropathy and Vestibular Areflexia Syndrome (CANVAS): a case series," *J. Neurol.,* vol. 269, *(10),* pp. 5668–5673, 2022.

[34] D. Pellerin *et al*, "Intronic FGF14 GAA repeat expansions are a common cause of ataxia syndromes with neuropathy and bilateral vestibulopathy," *Journal of Neurology, Neurosurgery & Psychiatry,* 2023.

[35] C. R. Gordon *et al*, "Bilateral vestibulopathy as the initial presentation of CANVAS," *J. Neurol. Sci.,* vol. 460, pp. 122990, 2024.

[36] V. Marcelli and B. Giannoni, "A Clinical Infrared Video-Oculoscopy Suppression Head Impulse (IR-cSHIMP) Test," *Audiology Research,* vol. 14, *(1),* pp. 151–165, 2024.

[37] A. Traschütz *et al*, "Sensory axonal neuropathy in RFC1-disease: tip of the iceberg of broad subclinical multisystemic neurodegeneration," *Brain,* vol. 145, *(3),* pp. e6–e9, 2022.
